# Active nematics

**DOI:** 10.1038/s41467-018-05666-8

**Published:** 2018-08-21

**Authors:** Amin Doostmohammadi, Jordi Ignés-Mullol, Julia M. Yeomans, Francesc Sagués

**Affiliations:** 1 The Rudolf Peierls Centre for Theoretical Physics, University of Oxford, Clarendon Laboratory, Parks Rd., Oxford, OX1 3PU UK; 2 Departament de Ciència de Materials i Química Física and Institute of Nanoscience and Nanotechnology, Universitat de Barcelona, Martí I Franquès 1, 08028 Barcelona, Catalonia Spain

## Abstract

Active matter extracts energy from its surroundings at the single particle level and transforms it into mechanical work. Examples include cytoskeleton biopolymers and bacterial suspensions. Here, we review experimental, theoretical and numerical studies of active nematics - a type of active system that is characterised by self-driven units with elongated shape. We focus primarily on microtubule–kinesin mixtures and the hydrodynamic theories that describe their properties. An important theme is active turbulence and the associated motile topological defects. We discuss ways in which active turbulence may be controlled, a pre-requisite to harvesting energy from active materials, and we consider the appearance, and possible implications, of active nematics and topological defects to cellular systems and biological processes.

## Abstract (web summary)

Active matter systems are made up of self-driven components which extract energy from their surroundings to generate mechanical work. Here the authors review the subfield of active nematics and provide a comparison between theoretical findings and the corresponding experimental realisations.

## Introduction

The term active matter describes natural or artificial systems that are out of thermodynamic equilibrium because of energy input to, or by, individual particles. Living entities such as birds, fish or bacteria intrinsically exist out of equilibrium by converting chemical content of their food into some form of mechanical work. Similarly, synthetic systems can be designed to perform work driven by energy from light or chemical gradients^1^. Active systems not only provide an experimental testing ground for theories of non-equilibrium statistical physics^2,3^, but also underpin the natural processes of life^4^. From pathological events such as biofilm formation or cell invasion to morphogenesis and even the flocking of fish, birds or animal herds, the physics of active matter plays a vital role.

An important feature of materials built from active entities is the emergence of collective motion, in which groups of active particles move together as a unit on scales that are significantly larger than the size of an individual. Everyday examples are the intricate patterns formed by airborne starling flocks or when a school of fish move together to avoid a predator. Similar collective behaviour persists down to micro-scales, where bacterial suspensions, tissues and intracellular filaments use their intrinsic activity to create motions on lengths larger than individual cells or proteins. There is increasing evidence that such collective behaviour is important in equipping the cells with an ability to invade and occupy their surrounding spaces or to shape various cell morphologies needed for tissue function^5^. Therefore, understanding the mechanism and dynamics of the collective motion of active materials is of considerable relevance to natural systems across a wide range of length scales.

Several theoretical and experimental model systems have been developed to study the collective behaviour of active matter. The goal is to mimic the behaviour of natural materials in a controlled manner to gain a better understanding of the mechanisms at work. One well-studied class of active matter which includes, for example, elongated bacteria and filamentous particles inside living cells can be simplified to a group of rod-shaped particles. These particles bear a resemblance to nematic liquid crystals, which contain elongated molecules characterised by long-range orientational order. The concept of ‘active nematics’ is raised to exploit and adapt the presently available theories that have been developed for liquid crystals over the decades. The main difference, however, lies in the ‘activity’ of the active matter, which leads to the spontaneous generation/annihilation of topological defects in active nematics, causing the destruction of long-ranged nematic order and thus the formation of chaotic-like features, namely ‘active turbulence’. For details, please refer to Box 1.

Here, we review the physics of active nematics describing experimental models and theoretical approaches. On the experimental side we will concentrate on microtubule (MT)—motor protein mixtures, but will also mention bacterial suspensions and cell layers. From the theoretical perspective, we will primarily focus on the continuum equations for active nematics and the extent to which theoretical and computational models can reproduce experimental observations. We will also describe the phenomenon of active turbulence, its relation to motile topological defects, and possible ways of controlling active turbulence by confinement or friction. Finally, we will discuss the relevance of active nematics to biological functionality.

### BOX 1 Active nematics and turbulence

Our understanding of active nematics draws heavily on decades of research into passive, molecular or colloidal nematic liquid crystals^6^, driven partly by their importance in display technology. Nematic particles are rod-like with head-tail symmetry. For some regions of temperature or concentration they can predominantly align in a given direction, termed the director, to give a nematic phase with long-range orientational, but no long-range positional order. The nematic phase is stabilised because it gives rise to an increase in free volume, and hence in entropy. Structural inhomogeneities in the material or external forcing can lead to conditions where there are mismatches between neighbouring domains that have different directions. This leads to topological defects, singularities in the orientation field. For a two-dimensional nematic liquid crystals two types of topological defect predominate: comet-like (+1/2) or trefoil-like (−1/2). The number associated with the topological defects, the winding number or topological charge, is the change in the orientation of the molecules around the singular points along a full 360° rotation: for +1/2 or −1/2 defects the molecules turn through +180 and −180, respectively. Unless they are pinned, +1/2 and −1/2 topological defects annihilate in pairs.
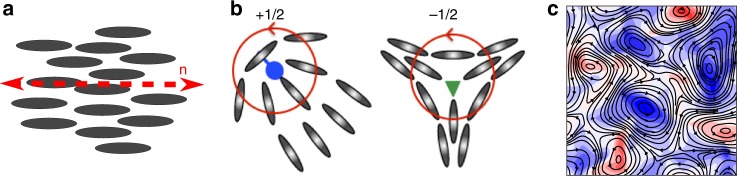


Activity destroys the long-range nematic ordering leading to active turbulence, characterised by short-range nematic order and chaotic flow fields with jets and swirls. Now topological defects can be created in pairs and, because the +1/2 defects are motile, they move away from each other.

Schematic representation of (**a**) nematic alignment along the director *n* and (**b**) comet-like (+1/2) and trefoil-like (−1/2) topological defects. **c** An example of active turbulence characterised by chaotic flows with vortices of different sizes. Black lines illustrate streamlines and the colourmap shows vorticity normalised with its maximum value, varying from +1 (red) for anticlockwise to −1 (blue) for clockwise vorticity.

## Active systems based on filamentary proteins and molecular motors

In vitro assays where biopolymers such as MTs or actin are mixed with, and subsequently driven by, motor proteins are valuable tools for deciphering crucial aspects of the cell machinery. This view was pioneered twenty years ago by Nédélec et al.^7^, who proposed a minimal realisation of the cytoskeleton as a system of stabilised MTs internally sheared by multi-headed kinesin molecular motors. By progressing along the MTs, the motors were able to organise them into an irregular lattice of asters (with the MTs arranged to point radially outwards) or vortex-like structures. This research was extended by using motility assays^8^. In their simplest version, these track the motion of filamentary proteins that are translocated by molecular motors whose tails are anchored on a rigid substrate. In a series of papers from the Bausch group, collective structures, in the form of actin clusters, swirls and bands, orders of magnitude larger than their constituents, were observed to move persistently in a high-density bath of actin driven by immobilised myosin motors^9,10^.

We now introduce an experimental system that will be central to the developments that we will review here. This was pioneered in the Dogic group^11^ and revisits the MT–kinesin mixture, adding a depleting agent, the non-adsorbing polymer polyethylene glycol (PEG) (see Box 2). The resulting attractive depletion interaction causes the highly concentrated, stabilised, fluorescently labelled MTs, which are of the order of 1.5 µm in length, to assemble into bundles, hundreds of microns long. The resulting aqueous-based, active gel is continuously remodelled by clusters of Streptavidin (Stv) and biotinylated kinesin (B-Kin), which undergo 8 nm steps along the MTs, fuelled by ATP hydrolysis^12^. By translocating between bundled MTs, kinesin clusters enhance their processivity—to about a micrometre for a single motor—before detaching.

Streptavidin-based clusters of (normally) two kinesins form a bridge between pairs of MTs and walk towards the plus ends. Hence, internal sliding of the filaments is driven by the relative polarity of the MTs in the assembled bundles. The kinesin cluster will walk towards the plus ends of an aligned pair without relative displacement of the MTs. By contrast, the processivity of the motors drives MTs of opposite polarity to slide past one another. Hence, kinesin clusters cause polarity sorting at a local level within isolated bundles, and eventually trigger their overall extension. In dense gels, bundle extension is followed by fracture, disintegration and fragment recombination, leading to a dynamical steady state as the system permanently struggles to attain a globally polarity-sorted configuration^13^. The network of bundled MTs is permeated by streaming flows whose spatial structure can be monitored by particle-tracking, revealing structured velocity–velocity correlation functions^11^.

Despite their tenuous textures, these active gels are very robust and their overall dynamics can be tuned by varying different control parameters, most importantly the activity, measured in terms of the ATP concentration, or the motor cluster, MT or PEG concentrations^13^.

### BOX 2 MT/motor protein mixtures

An experimental system that continues to be very important to developing the understanding of active nematics is a mixture of MTs and two-headed molecular motors. **a** Fluorescently tagged MTs from polymerised tubulin are brought together by the depleting action of PEG, and are cross-linked by clusters of B-Kin and Stv, resulting in active extensile MTs bundles in an aqueous suspension (**b**). As the motors walk along the MTs the bundles extend, are pushed apart, and re-form. **c** The active nematic self-assembles at the water/oil interface and gives rise to active turbulence for as long as there is sufficient ATP to fuel the motors.
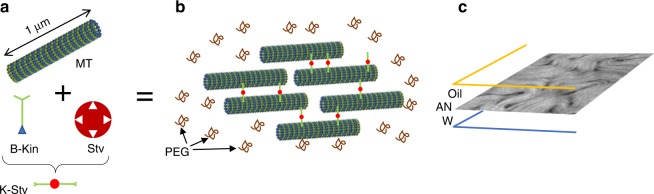


Schematic of the experimental system.

### Active nematic turbulence

**Fig. 1:**
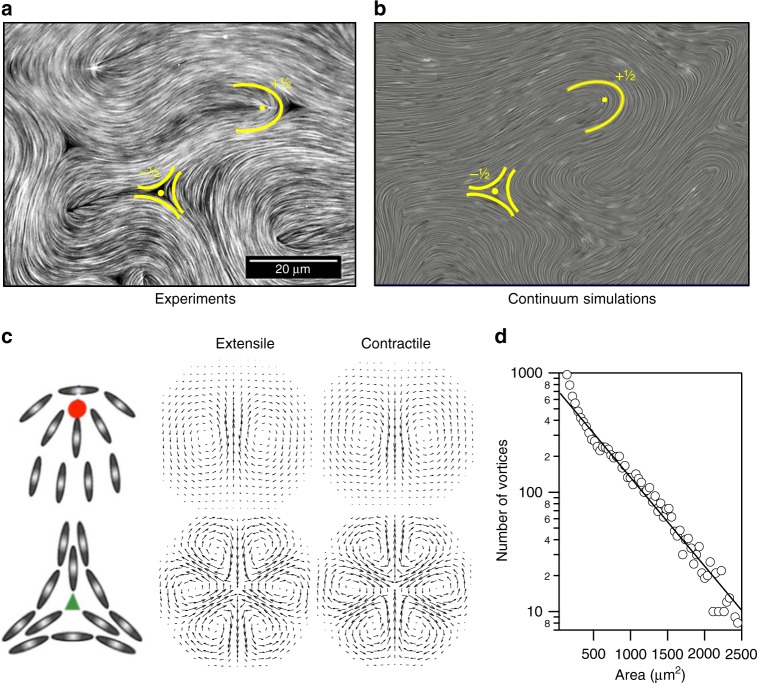
Active nematic turbulence. **a** Fluorescence confocal microscopy micrograph of the active nematic in contact with an oil of 0.05 Pa s (see Supplementary Movie 1 where positive defects are tracked). **b** Snapshot of the time evolution from solving the continuum equations of motion, showing active turbulence. A comet-like, +1/2, and a trefoil-like, −1/2 defect are highlighted in each case. **c** Particle alignment and velocity fields around ±1/2 topological defects in extensile and contractile active systems. **d** Experimental distribution of vortex sizes in an active nematic in the regime of active turbulence, adapted from data in ref. ^15^, Nature Publishing Group. The solid line is an exponential fit to the data

In the first experiments on the MT–kinesin system^11^ the mixture was placed in contact with an oily phase through a PEG-stabilised interface (Box 2). The active material progressively accumulates at the boundary, forming a 2D layer with aligned bundles displaying the head–tail symmetry of a nematic. The bundles continually extend and fold to form a dynamical steady state, an experimental realisation of what has come to be known as active turbulence because of its visual resemblance to turbulent fluid flow (Fig. 1a–d). Figure 1a shows a snapshot of the MT configuration in this state and Supplementary Movie 1 shows the dynamics. Behind its apparent disorder, this regime displays a distinctive spatial coherence. Indeed, a length scale can be easily identified in the scaling of the exponential distribution of vortex sizes, as predicted from numerical simulations^14^ and confirmed experimentally (Fig. 1d)^15^. We shall term this the active length scale: typically, it is of the order of tens of microns, i.e., a few tens of the length of the MTs.

It is apparent from Fig. 1a (see also Supplementary Movie 1) that the active gel is punctured by regions devoid of MTs. These can be identified as the cores of topological defects, a defining feature of nematic materials^16^ (see Box 1). In passive nematics the defects slowly anneal out unless trapped by imperfections or boundaries. A key difference in active nematics is that the active driving can provide energy to create topological defects^11^. They are formed in pairs of topological charge ±1/2 as a bundle bends, and then separate: at positive defects the local configuration of MTs resembles a comet whereas at negative defects it is star-like (Fig. 1c). The pairs of ±1/2 defects annihilate when they meet so a steady-state population of defects is achieved, with density depending on the activity and material characteristics of the active sample, and on the rheological properties of the hosting interface^17^. In active turbulence, the motion of topological defects appears to show chaotic trajectories. However, recent experimental observations on centimetre-scale samples, accumulating statistics for the orientation of thousands of comet-like (positive) defects over long-times, report the emergence of a system-spanning nematic order in the orientation of defects^18^. This somewhat debated issue has also been addressed using numerical simulations and theoretical analysis^19–24^.

Considerably before active turbulence was observed in MT-based systems, it had been reported in a biological multicellular context. The earliest examples are the bioconvection plumes formed by dense suspensions of algal cells or *Bacilus subtilis* bacteria^25^. Working with open drops of dense, aerobic *Bacilus subtilis* suspensions Dombrowski et al.^26^ observed large scale flows which they termed zooming bionematics^27^. It was shown later by Wolgemuth^28^ that the dynamics of such zooming bionematics can be captured by a two-phase model for a bacteria and fluid mixture. Active turbulence has also been observed in swarming sperm cells^29^, human bronchial epithelial cells^30^, and Madine–Darby canine kidney cells^31,32^.

## Theoretical models

A model that has been particularly successful in describing active nematics is a continuum theory that depends only on the symmetries of the system^19,33–38^ The method is based on equations of motion for a coarse-grained order parameter describing the orientation field, **Q**, and the velocity field, **u**.

Nematics have no long-range positional order, but do have orientational order that can be described by the director field **n** with (**n** = −**n**) to reflect the head–tail symmetry characterising the nematic state. While the director gives the orientation of alignment it carries no information about the magnitude of the ordering and it is useful, particularly when dealing with topological defects, to use an order parameter which is a traceless tensor ${\bf{Q}} = \frac{d}{{d - 1}}q\left( {{\mathbf{nn}} - {\mathbf{I}}{\mathrm{/}}d} \right)$, where *q* is the magnitude of the order, *d* is the dimension of space, and **I** is the identity matrix.

The dynamics of **Q** can be described by the nematodynamic equation^39^

1 $$\partial _t{\bf{Q}} + {\bf{u}} \cdot \nabla {\bf{Q}} - {\bf{S}} = \Gamma {\bf{H}}.$$

In addition to advection by the flow, elongated particles will respond to gradients in the flow. This is accounted for by the co-rotation term,

2 $$\begin{array}{ccccc}\\ {\bf{S}} = \left( {\lambda {\bf{E}} + {\bf{\Omega }}} \right) \cdot \left( {{\mathbf{Q}} + \frac{{\bf{I}}}{3}} \right) + \left( {{\mathbf{Q}} + \frac{{\mathbf{I}}}{3}} \right) \cdot \left( {\lambda {\mathbf{E}} - {\bf{\Omega }}} \right)\\ \\ - 2\lambda \left( {{\mathbf{Q}} + \frac{{\mathbf{I}}}{3}} \right)\left( {{\mathbf{Q}}:\nabla {\mathbf{u}}} \right),\\ \end{array}$$

where **Ω** and **E** are the vorticity and the rate of strain tensors, respectively. The relative dominance of the rate of strain and the vorticity in affecting the alignment of particles with the flow is characterised by the tumbling parameter λ.

The Γ**H** term describes relaxational dynamics of the nematic tensor to the minimum of a free energy through a molecular field defined as

3 $${\mathbf{H}} = - \frac{{{\delta} {{\cal F}}}} {{{\delta} {\mathbf{Q}}}} + {\frac{{\mathbf{I}}} {3}} {\mathrm{Tr}} \left( {\frac{{{\delta} {{\cal F}}}} {{{\delta} {\mathbf{Q}}}}} \right),$$

with Γ the rotational diffusivity. The free energy is typically taken as^6^

4 $${{\cal F}} = {\frac{A}{2}} {\mathbf{Q}}^{2} + {\frac{B}{3}} {\mathbf{Q}}^{3} + {\frac{C}{4}} {\mathbf{Q}}^{4} + {\frac{K}{2}} \left( {\nabla} {\mathbf{Q}} \right)^{2},$$

where the coefficients of the bulk terms *A*, *B*, and C are material parameters, and the final term describes the elastic free energy cost of spatial inhomogeneities in the order parameter field, assuming a single elastic constant *K*.

The order parameter dynamics is strongly coupled to the fluid velocity through the advection and co-rotation terms in Eq. (1). The velocity field, assuming a constant density *ρ*, obeys the incompressible Navier–Stokes equations:

5 $$\nabla \cdot {\mathbf{u}} = 0,$$

6 $$\rho \left( {\partial _t{\mathbf{u}} + {\mathbf{u}} \cdot \nabla {\mathbf{u}}} \right) = \nabla \cdot {\mathbf{{\Pi}}},$$

where **∏** is a general stress tensor, which includes the pressure, *P*, and the viscous, elastic, and active stresses:

7 $${\mathbf{\Pi}}^{{\mathrm{viscous}}} = 2\eta {\mathbf{E}},$$

8 $$\begin{array}{ccccc}\\ {\mathbf{{\Pi}}}^{{\mathrm{elastic}}} = - P{\mathbf{I}} + 2{\mathrm{\lambda }}\left( {{\mathbf{Q}} + {\mathbf{I}}{{/}}3} \right)\left( {{\mathbf{Q}}:{\mathbf{H}}} \right)\\ \\ - \lambda {\mathbf{H}} \cdot \left( {{\mathbf{Q}} + \frac{{\mathbf{I}}}{3}} \right) - {\mathrm{\lambda }}\left( {{\mathbf{Q}} + \frac{{\mathrm{I}}}{3}} \right) \cdot {\mathbf{H}}\\ \\ - \nabla {\mathbf{Q}}\frac{{\delta {\cal F}}}{{\delta \nabla {\mathbf{Q}}}} + {\mathbf{Q}} \cdot {\mathbf{H}} - {\mathbf{H}} \cdot {\mathbf{Q}},\\ \end{array}$$

9 $${\bf {\Pi}}^{{\mathrm{active}}} = - \zeta {\mathbf{Q}}.$$

In these equations, *η* is the viscosity and *ζ* is the activity coefficient, which sets the strength of the stresses generated by active particles. In the absence of activity (*ζ* = 0), Eqs. (1), (5), and (6) correspond to the nematohydrodynamic equations of motion for passive nematic liquid crystals^39^.

The proportionality of the active stress to the nematic tensor, Eq. (9), comes from coarse-graining the dipolar flow fields that are generated by each of the active particles^25,40,41^. Depending on the sign of the activity coefficient, *ζ*, two general modes of self-propulsion of particles can be distinguished: *ζ* > 0 describes extensile (pusher) particles which drag the fluid towards their sides, pushing it away along their elongation axis, while *ζ* < 0 corresponds to contractile (puller) particles that pull the fluid in along their length and expel it from their sides. MT–kinesin mixtures are extensile^11^, whereas actomyosin gels are contractile^42^.

Despite the large number of parameters, the governing equations of active nematics can be significantly simplified for extensile systems of microscopic particles. For such systems, due to their small size ~0(μm) and velocities ~0(μm/s), inertial effects are negligible (Re ≈ 0) and the lhs of Eq. (6) can be neglected. Moreover, in MT–kinesin mixtures the time scale of energy injection is typically orders of magnitude faster than the elastic relaxation of the nematic orientation, indicating that active stresses dominate elastic stresses. Under this condition, it can be shown^43^ that extensile activity can itself lead to an effective bulk free energy and active turbulence even if the bulk free energy, i.e. the material constants *A, B,* and *C* in Eq. (4), are put to zero. Therefore, for an extensile system of sufficiently small particles such as MT/kinesin mixtures the governing equations of active nematics can be simplified and written in dimensionless form as:

10 $$\frac{{\tilde D{\mathbf{Q}}}}{{\tilde Dt}} - {\tilde{\mathbf S}} = \frac{1}{{{\mathrm{Pe}}}}\tilde \nabla ^2{\mathbf{Q}},$$

11 $$\tilde \nabla \cdot {\tilde{\mathbf u}} = 0,$$

12 $$0 = \tilde \nabla \cdot \left( {{\tilde{\mathbf {\Pi}}}^{{\mathrm{viscous}}} - \frac{1}{\chi }{\tilde{\mathbf {\Pi}}}^{{\mathrm{active}}}} \right).$$

As a result, the parameter space is significantly reduced to two dimensionless groups, $\chi = \eta U/\left( {\zeta l_Q} \right)$ and ${\mathrm{Pe}} = Ul_Q/\Gamma K$, where we have introduced a characteristic velocity *U* and a characteristic length *l*_*Q*_. Together with the tumbling parameter, these constitute the relevant variables for describing a MT–kinesin motor mixture.

The continuum equations have been extended to two-phase^44^ and viscoelastic^45^ flows of active nematics by adding a concentration field or a polymer conformation tensor as additional order parameters, respectively. These calculations identify oscillatory, shear banded states, and active turbulence even in the limit of high polymer density^45^.

An alternative approach, based on kinetic theory, has also recently been used to model the nematohydrodynamics of active, rod-like particles^46^. Each particle is represented as a slender rod with a surface velocity that results in extensile or contractile dipolar flows, and the distribution function for the number density of the particles is described by the Smoluchowski equation. The particle concentration and nematic tensor are then constructed from the first and second moments of the distribution function, and the centre of mass position and orientation of the rods are found from slender body theory for zero-Reynolds number flows. Such a kinetic theory approach is able to reproduce the generation of active turbulence and the dynamics of active defects.

While the continuum approach provides insights into the dynamics and pattern formation on scales greater than that of individual active particles, it does not capture the details of particle-particle interactions or the physical properties of individual active particles such as their bending rigidity. On the other hand, while kinetic approaches allow for building models from detailed microscopic properties, they come at the cost of additional complexity. New model parameters are introduced, implementation of variable boundary conditions becomes challenging, and numerical simulations are typically slower than for continuum models.

Moreover, none of the approaches discussed above account for any flexibility of active filaments. Recent microscopic simulations of semi-flexible active polymers in the absence of hydrodynamic interactions, have shown that flexibility of filaments leads to renormalisation of the effective bending rigidity of the system^47^. This suggests that the effect of flexibility might be absorbed as a renormalisation of elastic constants in the continuum theory. Indeed, recent work has begun to use the velocity and structure of topological defects to map material constants such as the orientational elasticity *K* in MT–kinesin mixtures^48^ and actin-based nematic materials^49^ to model parameters.

### Results from continuum simulations of active turbulence

Numerical solutions of the active nematohydrodynamic Eqs. (1)–(9) result in a state very similar to the active turbulence seen in the experiments on MTs and kinesin motors (see Fig. 1a, b for a comparison between numerical solutions and experimental data). Hence, they have helped to highlight the roles of nematic symmetry, flow, activity and elasticity in understanding the out-of-equilibrium physics.

Linear stability analysis of the equations shows that for any non-zero activity, a 2D extensile (contractile) active nematic is unstable to bend (splay) deformations of the director field^40,50.51^. Thus, in the absence of boundaries, the nematic state is unstable to any level of activity: the dominant unstable mode grows linearly with activity. Linear stability analysis cannot predict the new state of the system, but numerical solutions of the equations of motion show that there is a crossover to active turbulence^35,36^. Measurement of the velocity-velocity correlation function^38,52^ leads to a decay, which closely mirrors that in the experiment and in a mean-field theory of active turbulence^14^, and shows that the active length scale which governs the decay of the vorticity correlations is $\sqrt {K/\zeta }$.

The distortions created by the hydrodynamic instabilities tend to localise to form ‘walls’, lines of high distortion separated by nematic regions^53^. Because of their high elastic energy the walls are preferential sites for the formation of ±1/2 topological defects. Rather than immediately annihilating, the +1/2 defect moves away from the −1/2 defect, preferentially along the wall, thus restoring nematic order which is then subject to further instability. When defects of opposite charge meet they annihilate, and the continual creation and annihilation leads to a steady state with a well defined defect density and spacing.

We stress that a significant distinction from passive nematics is that activity results in a self-propulsion speed for +1/2 defects^35,36,54,55^ as shown in Fig. 1c. This can be understood from Eqs. (6) and (9), which show that the large gradients in **Q** around the defect generate unbalanced forces and hence a flow field. −1/2 defects also generate stresses but these are usually close to balanced because of the symmetry of the corresponding director field^54,56^ (Fig. 1c). Defect motion provides a good way of distinguishing extensile and contractile materials: for extensile systems comet-like +1/2 defects move towards their ‘head’, as has been shown experimentally for MT bundles^11^, human bronchial epithelial cells^30^ and Madine–Darby canine kidney (MDCK) cells^32^, whereas for contractile systems they move towards their ‘tail’, which has been observed in experiments on mouse fibroblast cells^57^. As we will see in the upcoming section, this self-propulsion property of +1/2 defects in active nematics plays an important role in the emergence of exotic patterns of motion.

We note that in active turbulence energy is input at the scale of individual particles, which should be contrasted to inertial turbulence where the energy is input at large scales. Indeed detailed statistical analysis of active turbulence, in experiments^30,58^, and in numerical simulations^59–61^ shows a clear distinction from inertial turbulence in terms of intermittency, energy spectrum and flow structure.

## Constraining active nematics

In this section we discuss a number of experimental and theoretical means of constraining the flows and thus stabilising defect trajectories in the otherwise chaotic motion characteristic of the active turbulence regime. One way of screening the hydrodynamic flows is by introducing friction. Here, by friction we mean momentum transfer between active fluids and their surroundings (as opposed to any friction between active particles). Momentum loss to the environment is indeed present in many active systems such as bacteria that are contacting a surface, or cells crawling on a substrate. We also discuss a viscous control method, where viscous dissipation in the fluid that is in contact with the active layer dampens the momentum of the active particles. Moreover, we describe confinement of active matter in both two- and three-dimensions as another way of constraining active turbulence.

### Substrate friction

The impact of frictional damping on the behaviour of active nematics has been investigated theoretically^19,21,22,62^. One way to include friction in the governing equations of motion is to add a damping term −*f***u**, proportional to velocity, to the left-hand side of the momentum equation (6). As friction is increased the dynamics of the active nematic slows down, leading to lower velocities and walls that persist for a longer time. Solving the continuum equations shows that the number of topological defects increases with increasing friction.

Frictional damping results in hydrodynamic screening of the flow over a length $L_{{\mathrm{sc}}} \sim \sqrt {\eta {\mathrm{/}}\rho f}$. As the friction is increased the frictional screening length becomes shorter until it matches the activity-induced length scale of the flow, $\sqrt {K{\mathrm{/}}\zeta }$. The numerics show that at this point the active turbulence can self-organise into a regular lattice of vortices accompanied by an ordered array of topological defects. This gives a potential route to produce positional ordering of nematic defects^19^.

Moreover, at very high friction the walls are stabilised and the defect number drops to zero^62^. Here, frictional damping dominates viscous dissipation of the momentum and, therefore, the active forcing is balanced by the friction. Under these conditions, the nematic approaches the so-called ‘dry’ limit, where there is no long-range transport of momentum^19,63^. Although the continuum description of the dry limit does not lead to formation of topological defects^64^, particle-based simulations of active self-propelled rods and semi-flexible active polymers without hydrodynamic interactions do produce defects^47,65^. The dry limit has been shown to reproduce the pattern formation in gliding bacterial cells such as soil bacteria M. xanthus, which can be considered nematic because they periodically reverse their direction of motion as they move across a surface^66,67^.

### Viscous control

**Fig. 2:**
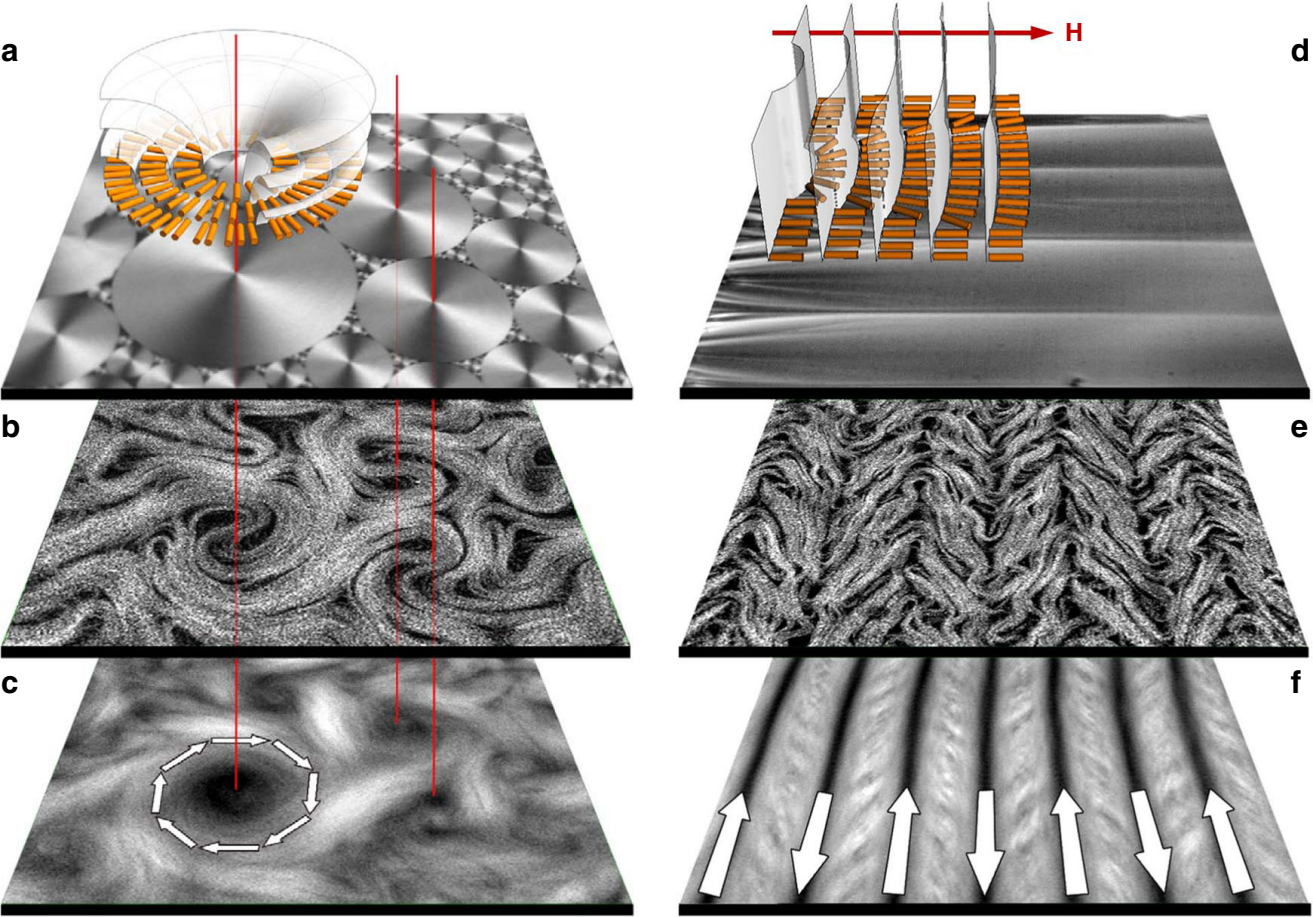
Alignment of an active nematic by anisotropic soft interfaces. In **a**–**c** the active nematic is in contact with an unconstrained smectic-A phase. In **d**–**f**, an external magnetic field (**H**) has aligned the smectic-A. In **a** and **d** the confocal reflection micrographs show the structure of the smectic-A phase at the interface with the aqueous phase in each case. The diagrams are sketches indicating the ordering of the smectic-A planes and liquid crystal molecules. **b**, **e** Fluorescence confocal micrographs of the active nematic layer with dynamical patterning that results from contact with the anisotropic interface. **c**, **f** Time averaged fluorescence confocal micrographs (total integration time 300 s). Arrows indicate the direction of the organised active flow. Field of view is 300 ×300 μm^15^^,68^. (Adapted with permission from Nature Publishing Group and AIP Publishing)

The possibility of controlling the active nematic by changing the properties of the viscous fluid lying next to the active layer (Fig. 2a) has been experimentally investigated in a series of recent publications^15,17,68^. The simplest possibility is to interface the active nematics with oils of different viscosity. An increase in oil viscosity leads to a larger number of topological defects in agreement with the simulations showing that the number of topological defects increases with friction^19^. This is because the active defects experience a higher resistance to flow and hence move more slowly. Thus their annihilating encounters becomes less frequent and their population increases.

Building on this work, recent experimental observations have described how active flows can be controlled by replacing the contacting isotropic oil with an anisotropic liquid crystal^15,68^. In a reference experiment using a passive nematic, octyl-cyanobipheny (8CB), there was no apparent feedback affecting flow in the active boundary layer, even when the orientation direction of the passive nematic was altered using a magnetic field.

However, when the 8CB entered the smectic-A phase (SmA), the striking observation was that the active flows partitioned into a set of circular swirls of different sizes, but above some minimum radius^15^. Swirls covered most of the interface, and were interspersed by persistent, apparently chaotic streams of flowing bundles (Fig. 2b, c). This occurs because the organisation of the active nematic is slaved to the structure of the contacting passive liquid crystal. At room temperature the SmA phase spontaneously self-assembles into polydisperse domains, known as toroidal focal conics^69^, which have circular footprints formed by concentric SmA planes (Fig. 2a) at the interface with the active layer. The 8CB molecules are radially oriented in concentric rings and the boundary shear stress experienced by the active filaments is much lower along than perpendicular to the rings. Hence, bundles preferentially move along circular trajectories, centred on the focal conics (Fig. 2b, c). The minimum swirl size, of the order of the active length scale, is explained by noting that a swirl must incorporate a topological charge +1 and so it must contain at least two +1/2 defects.

When a moderate magnetic field is applied, the liquid crystal with positive magnetic anisotropy organises into a structure with flat smectic planes perpendicular to the field, and hence the easy flow direction must be along the magnetic driving (Fig. 2d). Experiments confirm this, showing that the turbulent nematic is regularised into parallel stripes of uniform width aligned perpendicular to the field (Fig. 2e, f). It is interesting to note that in both the circular and linear self-organisation the velocity of the laminar flow is proportional to the activity^68^. This should be contrasted with the turbulent state where the theoretical prediction is $v \sim \sqrt \zeta$^14^.

### Two-dimensional confinement

**Fig. 3:**
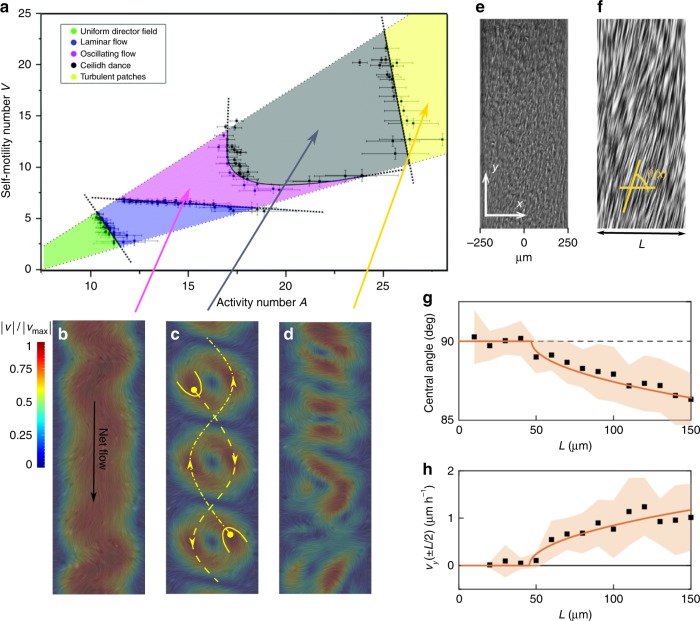
Flow states in active nematics confined to a 2D channel. **a** Regions of stability of different flow states as a function of the activity number, $A = L\sqrt {\zeta /K}$, which characterizes the ratio of the channel width *L* to the active length scale $\sqrt {K/\zeta }$, and a dimensionless +1/2 defect self-motility *V*, obtained by solving the continuum equations of active nematics^70^ (adapted with permission from Royal Society of Chemistry). **b**–**d** Representation of the flow states. The flow is confined by lateral walls in the horizontal direction. The colourmap corresponds to the normalized magnitude of the velocity and the yellow lines illustrate the trajectories of +1/2 defects within the channel. **e**, **f** Experimental realisation of laminar shear flow in a confined monolayer of fibroblast cells:^74^ cell orientations by phase contrast (**e**) and the line integral convolution method (**f**). **g**, **h** Increasing the channel width leads to an active Fréedericksz transition from a no flow to a spontaneous flow state as predicted by continuum active gel theory^50^. The comparison of the experimental measurements (black squares) with the analytical predictions (orange solid lines) shows excellent agreement both for the (**g**) central angle and (**h**) the velocity (*v*_*y*_) of the flow at the mid region of the channel^74^. (Adapted with permission from Nature Publishing Group)

The hydrodynamics of active nematics can also be screened by physical confinement. By analogy to frictional screening one might expect stabilising effects as the size of the confinement becomes comparable to the intrinsic active length scale. Numerical simulation of the continuum equations^70^ shows that, by confining an active nematic within an infinitely long two-dimensional channel, arrays of equi-spaced, opposing velocity vortices are stabilised in this regime (Fig. 3a–d).

The defect dynamics in this state are surprising. Elastic interactions attract the −1/2 defects to the boundaries of the channel. The +1/2 defects, however, are self-propelled. As a result of their hydrodynamic interaction with the vortices and the channel walls, they form two lines, moving in different directions through the channel on sinusoidal trajectories^70^. This state has been termed the ‘Ceilidh dance’ in analogy to the Grand Chain in Scottish country dancing (Fig. 3c).

As the activity is increased the vortex size becomes smaller than the channel width (Fig. 3d). This leads to co-existence between lengths of ordered vortex-lattice and disordered turbulent patches, with the fraction of the channel occupied by turbulence increasing with increasing activity. The turbulent patches, formed by fluctuations, can split and decay. This behaviour is reminiscent of directed percolation, and numerical simulations measuring critical exponents confirm that the transition belongs to the directed percolation universality class^71^. Similar behaviour has been reported in numerical and experimental studies of the transition to inertial turbulence in driven pipe or Couette flows^72,73^.

Conversely, if the activity is decreased, so that the vortices are too big to form in the channel, the flow oscillates, and then settles down to laminar (Fig. 3b). Further decrease in the activity results in a quiescent state. The transition occurs at a threshold $\zeta _c \propto 1/L^2$, which goes to zero for a two-dimensional, unbounded system ($L \to \infty$)^50^. This transition from no flow to spontaneous flow, first predicted theoretically by Voituriez et al.^50^, has very recently been confirmed experimentally by observing the spontaneous formation of shear flows in confined mouse fibroblast cells^74^ (Fig. 3e–h).

We note that, in addition to the activity and the size of the confinement, the anchoring condition of the directors on the boundaries could play a role in determining the flow states in confined active nematics. Such a dependence on anchoring condition has been reported, both theoretically and numerically^34,75,76^ in rectangular confinements.

Defect motion can also control the dynamics of active nematics confined to a circle^46,70,77–80^. In a circular geometry, planar or homeotropic boundary conditions introduce a + 1 topological defect. This splits into two +1/2 defects, which act as local pumps for the flow as observed in experiments on mouse fibroblast cells in circular confinement^57^. Simulations show that the defects are driven towards opposing walls where they can remain pinned and stabilise arrays of flow vortices or continue to move, along periodic closed orbits. They can also be absorbed by the walls and then recreated in a manner similar to the production of defects in active turbulence, giving rise to circular flows. Periodic flows of four defects have also been reported and, as the size of the container is increased, active turbulence is restored^46,80^ Somewhat surprisingly, the recent numerical work shows that in circular confinement flow states are insensitive to the boundary conditions for the director^80^. It is suggested that the hydrodynamic flows created by topological defects screen the anchoring effect, leading to a bulk region inside the circular geometry where the alignment at the boundaries is not felt by the active particles^80^.

### Three-dimensional confinement

The ability to use geometrical constraints to switch between different patterns of collective motion, and in particular to obtain coherent flows, suggests the possibility of devising active micro-fluidic devices. As a first step in this direction, recent experiments^81^ have shown that confining a MT–kinesin active gel in a three-dimensional micro-channel can produce long-range coherent flows up to metre scales. Surprisingly the transition to directed flow appears to depend on a scale-independent criterion related to the aspect ratio of the micro-channel, and not on the active length. The mechanism of the flow regularisation is not fully understood, but the authors^81^ suggest that it may be related to layers of aligned active nematic on the walls of the micro-channel that power the whole fluid.

In addition, oily emulsions of active droplets were first reported in Sanchez et al.^11^. A more specific study considering active vesicles has been published more recently^82^. In both cases the effects reported refer to the action of flows of active material which resides on a spherical shell at the inner border of the aqueous/surfactant interface. For moderately small droplets, active turbulence is suppressed, but now the minimum set of active defects is four +1/2 elements. This is a consequence of the Gauss–Bonnet theorem that, for this geometry, demands the existence of a combined defect charge +2^83,84^. For moderate activities, the defects follow closed trajectories that quasi-periodically oscillate between tetrahedral and coplanar arrangements.

The experiments have inspired several modelling approaches, which reproduce the observed defect dynamics^85–87^. Recent simulations have also extended the geometry of the shell to prolate and oblate spheroids, showing that the interplay between attraction of the +1/2 defects to the maxima of Gaussian curvature, and repulsion between the defects, can lead to distinct patterns of spatial organisation which depend on the shape of the shells^88^. On prolate spheroids two of the defects are localised at the poles and the other two oscillate around the waist of the shell, while on oblate spheroids defects can undergo relative rotational motion, reminiscent of Ceilidh dynamics. It has also been shown experimentally^48^ that constraining mixtures of MT–kinesin motors on a torus-shaped shell can lead to localised defect unbinding at regions of opposite Gaussian curvature.

The effects of confining cytoskeletal material within a droplet have also recently been studied using Xenopus egg extracts^89^. When the extracts were encapsulated in droplets, the extensile bundles pushed against the droplet boundary leading to bundles aligning into a rotating vortex structure. In addition, reconstituted contractile gel systems comprising F-actin and myosin have been used to study the effects of boundaries on shape changes and the dynamics of global contraction^42^.

## Active defects in cell biology

The recognition of topological defects in biological systems is rooted in the observations of Elsdale^90^ on the orientational order of fibroblast cells and the experiments of Benhoeffer and Grinvald^91^ who demonstrated that the orientation maps of cells in the cat’s visual cortex are characterised by patches of cells, encircling centre points, termed ‘pinwheel’ structures. The pinwheels play an important role in the organisation of neurons in the visual cortex and in the response to visual stimuli. The analogy to liquid crystals showed that they are indeed ±1/2 topological defects and theories based on the simple orientational dynamics of passive nematics faithfully reproduced the organisational patterns of pinwheels observed in the experiments^92,93^.

Soon after this, pioneering experiments by Gruler et al.^94,95^ described the analogy between nematic liquid crystals and amoeboid cells. They showed that nematic ordering and topological defects emerge in dense assemblies of melanocytes (cells located in skin, eye, inner ear, bones and heart), fibroblasts (cells important for tissue maintenance and wound healing), osteoblasts (cells that synthesise bone) and lipocytes (fat cells). By estimating the orientational elastic constants from defect structures in melanocyte cells, they showed that resistance to bend deformations is stronger than to splay (*K*_splay_ < *K*_bend_), and they demonstrated that, in analogy to conventional liquid crystals, the nematic director can be guided by creating parallel scratches on the cell substrate. More recently, experiments on mouse fibroblast cells have demonstrated long-range nematic order and topological defects that become trapped between nematic domains as the density of the cells increases and the tissue reaches a frozen state^96^. In particular, confining fibroblast cells within a circular geometry^57^ results in two +1/2 defects at long times.

The analogy to nematics is not specific to eukaryotic cells, and it has been extended to prokaryotic cells such as bacteria. A growing biofilm of *E. coli* bacteria in a micro-channel undergoes a transition from an isotropic to a nematic phase as the individual cells divide and the colony expands^97^. Similarly, recent experiments have shown that expanding colonies of bacteria are characterised by nematic domains in the form of ‘mosaic-like’ structures^98^. Indeed, numerical simulations of growing active nematic colonies have predicted that the self-propelled motion of +1/2 defects leads to the formation of finger-like protrusions and can induce morphological changes to the shape of the colonies^99^.

**Fig. 4:**
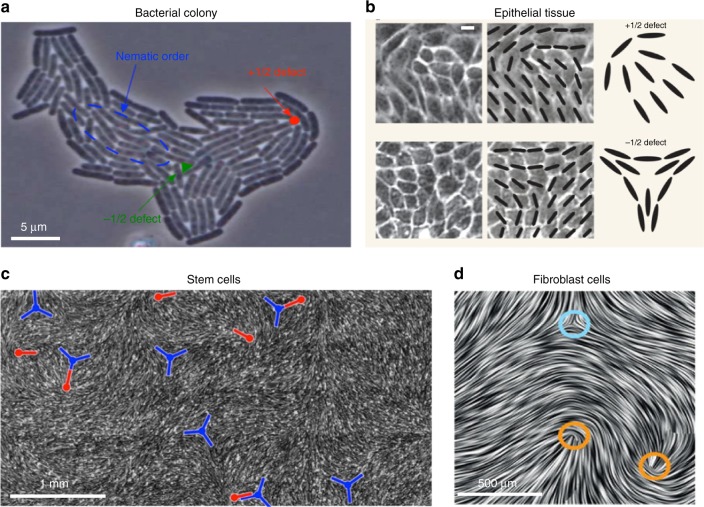
Active nematic defects in biological systems. **a** Growing colony of *E. coli* bacteria^99^ (Copyright (2014) by the American Physical Society). The motion of +1/2 defects towards the growing interface can lead to shape changes of the colony. **b** Epithelial tissue of Madine–Darby canine kidney (MDCK) cells. Scale bar is 10 μm^32^ (Nature Publishing Group). Strong correlations between the position of +1/2 defects and cell death and extrusion have been reported. **c** Monolayer of neural progenitor stem cells^100^ (Nature Publishing Group). Cells are depleted from −1/2 defects (blue, trefoil symbols) and accumulate at +1/2 ones (red, comet-like symbols). **d** Dense monolayer of mouse fibroblast cells^57^ (Nature Publishing Group) showing −1/2 and +1/2 topological defects marked by blue and orange circles, respectively

In these examples the long time behaviour is mainly characterised by the passive, elastic properties of the cells, since their activity is dominated by strong substrate friction. However, very recent work has identified biological systems where topological defects are continually created as in active nematics. Examples are shown in Fig. 4. Strikingly, there have also been suggestions that the active nematic characteristics may be related to biological functionality^32,100,101^.

**Fig. 5:**
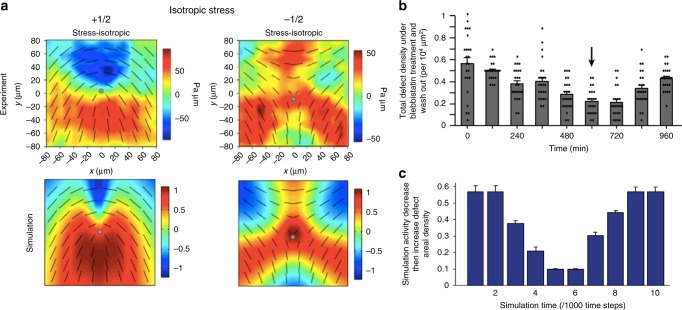
Stress patterns and density of topological defects. **a** Comparison of the isotropic stress around +1/2 (left) and −1/2 (right) topological defects between the experiments on monolayers of MDCK cells and continuum numerical simulations of active nematics. Colourmaps show the magnitude of the isotropic stress with blue (red) corresponding to mechanical compression (tension). **b**, **c** Total defect areal density evolution as a function of time. In the experiments (**b**) blebbistatin was introduced at *t* = 0 to suppress the cell motility and was washed out at the time 600 min (shown by an arrow). Similarly in the simulation (**c**) the activity parameter decreased at simulation time *t* = 0, then increased at *t* = 5^32^. (Nature Publishing Group)

Epithelial cells are tightly connected by means of cell–cell junctions and activity constantly drives deformation of the shape of the cells. By mapping out the (coarse-grained) direction of the long axis of the deformed cells, Saw et al.^32^ identified nematic order and motile topological defects within a two-dimensional confluent layer of epithelial cells (Fig. 4b). Continuum active nematic theories faithfully predict the flow fields and stresses around the defects (Fig. 5a), and, in agreement with the theory, experiments show that there is a correlation between the level of activity (controlled in the experiments by adding blebbistatin) and the number of defects in the cell layer (Fig. 5b, c).

Unexpectedly, the experiments revealed a strong correlation between +1/2 topological defects and sites of apoptosis, where a cell dies and is then expelled from the tissue monolayer. This is linked to the relatively high compressive stress at the head region of +1/2 defects. The compressive stress triggers a mechanotransductive response within the cell (movement of the protein YAP from nucleus to cytoplasm), which triggers cell death and a simultaneous expulsion of the cells at +1/2 defect sites. The authors showed that microcontact printing of the substrate could be used to control the number of topological defects, and hence the rate of apoptosis, in different areas of the confluent cell layer.

Kawaguchi et al.^100^ worked with neural progenitor cells (Fig. 4c), showing that, at high densities and under confinement, they are capable of aligning over large length scales, forming migratory streams resembling those observed in adult populations. The cells showed a clear tendency to deplete the neighbourhood of −1/2 defects and instead to accumulate at +1/2 defects, forming 3D ‘mounds’. Friction between the cells and the substrate was suggested as a potential mechanism for this behaviour; however the reason for the formation of the mounds is not yet fully explained.

Another system which demonstrates that motile particles are attracted to +1/2 defects is ‘living liquid crystals’. These are bacteria dispersed in aqueous-based liquid crystals^102^, a set-up which allows the swimming characteristics of the bacteria and the orientational order of the medium to be controlled independently^103,104^. The bacteria align along the director of the liquid crystal and motility is tuned by the amount of dissolved oxygen. After an oxygen supply is initiated, bacteria start swimming as pushers and trigger a stripe-like instability that, at sufficiently high activity, gives rise to the nucleation of half-integer defects proliferating into active turbulence. Furthermore the experiments show that pre-imposed topological defects in the passive liquid crystal can control bacterial concentration by depleting the bacteria from the −1/2 defects and attracting them to the +1/2 ones^103^.

## Perspectives and future directions

In this review, we have summarised the current understanding of active nematics, from both the experimental and the theoretical points of view, and highlighted the most recent advances in this rapidly growing field. In particular, we have focussed on active turbulence, a state characterised by high vorticity and the creation of motile topological defects. Given the increasing number of experimental systems and models that are expanding our knowledge of active nematics, several new directions can be envisaged.

Geometrical confinement is a promising direction for stabilising the otherwise chaotic motion of active particles and channelling them into useful flows. Beyond MT-based systems, the possibility of inducing persistent circular currents by confining confluent epithelial cell sheets was reported some time ago^78^. Similar circular flows have been observed in dense bacterial suspensions confined within circular geometries^105^. This work has been extended to lattices of circular cavities each containing a bacterial vortex and connected by short channels. Coupling between the cavities led to a chequerboard pattern of clockwise and anti-clockwise vortices^106^. Active suspensions have been used to drive submerged microscopic gears^107–109^ and the possibility of persistent correlated rotation of an array of discs surrounded by an active fluid has been demonstrated numerically^110^. Viscous control has been used to pattern flows in active nematics^15,17,68^ and a similar principle might be applied to active biological materials such as bacterial biofilms or, much more of a challenge, to whole cell ensembles with the perspective of steering the natural patterns of growing tissues. However, the design and development of active micromachines is still in its infancy.

Up to now, most of the research on active nematics has been limited to two dimensions. Numerical simulations of active nematics in 3D channels show that the topological defects form lines and loops^111^ as in passive liquid crystals^6^, but it is not yet clear how activity modifies the behaviour and properties of these disclination lines. Moreover, whether the current understanding of 2D active nematics, such as defect velocities and scalings of velocity or vorticity correlation lengths, can be extended to 3D is unexplored.

**Fig. 6:**
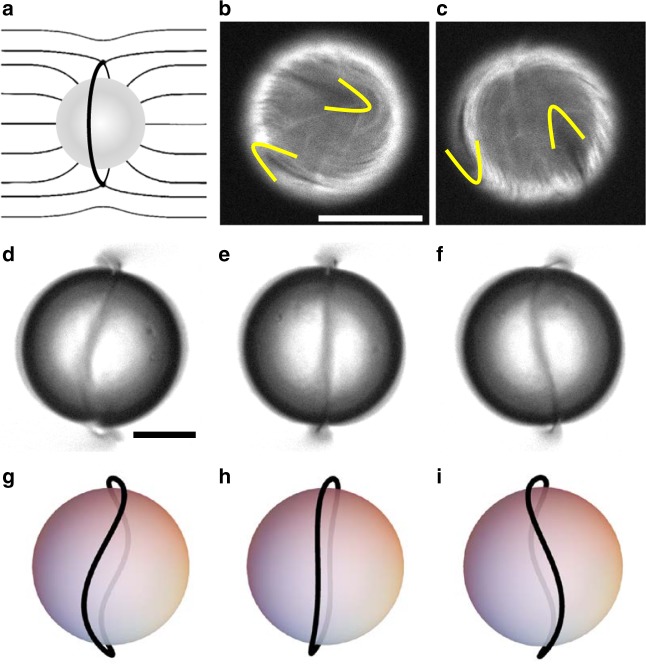
Active shells embedded in a nematic liquid crystal. **a** A spherical inclusion in a homogeneous nematic liquid crystal leads to the formation of an annular disclination (Saturn Ring). **b**, **c** Fluorescence micrographs showing the evolution of an active nematic spherical shell with four +1/2 defects (two visible at a given time), marked with yellow contours. **d**–**f** Brightfield micrographs with the oscillations of the Saturn Ring that has formed around an active shell. **g**–**i** Sketches showing the structure of the Saturn Ring in the micrographs (**d**–**f**). Scales are 20 μm^111^. (Adapted with permission from American Association for the Advancement of Science)

An interesting system that is just beginning to be explored experimentally is an active nematic emulsion, i.e., active droplets emulsified in a passive nematic phase, described in Fig. 6^112^. New aspects here would be the possibility of exploring coupling between active and passive defects or the behaviour of ensembles of active droplets. One could envisage the liquid crystal elastic matrix mediating new routes of dynamic self-assembly fuelled by the intrinsic activity of the individual units or favouring some sort of synchronisation of individual active flows into large scale collective dynamic modes.

Even in two dimensions there are still a number of key unanswered questions. The relative roles of thermodynamic interactions and the dipolar form of the far flow fields in determining the nematic symmetry of the active system is not yet clear. It is also not fully understood why some systems, such as MDCK or HBE cells, show characteristics of extensile activity^30,32^ while others, such as fibroblast cells, are contractile^74^. Moreover, there are questions about how individual polar entities can create nematic groups. It is widely accepted that, unlike an individual MT bundle, a single swimming bacterium or an isolated epithelial cell is polar. Yet, in a dense bacterial suspension or an epithelial monolayer, half-integer (nematic) topological defects are observed rather than the full integer defects expected from polar symmetry. One could conjecture that the nematic symmetry arises from the coarse-graining of velocity fields at the multicellular level, averaging out any preferred direction. However, multiscale approaches accounting for both individual and multicellular dynamics are needed to pinpoint the mechanisms behind this crossover and to bridge the gap between the continuum and more microscopic theories. Recently it has been demonstrated that small modification of the alignment mechanism between active particles can even lead to a coexistence of nematic and polar patterns^113^.

Moreover, it is not clear how activity or orientational elasticity, the two parameters that determine the active length scale, should be measured in experimental systems. In in-vitro MT/kinesin systems, activity seems to be directly related to the chemical potential of ATP, but orientational elasticity depends on the concentration of depletant, motors and MTs in a non-trivial way^11^. In addition, the extent to which hydrodynamics plays a role in some of the biological active nematics such as cell monolayers deserves further investigation. Measurements of velocity fields of various kinds of cells around topological defects^30,32^ show very good agreement with predictions from active nematohydrodynamic equations. However, it is not obvious whether long-range hydrodynamic interactions are present in such systems.

Finally it is exciting to view biological systems as active materials, in line with the increasing emphasis on mechanics as a regulator of cellular functions^5^. For example stress can cause proteins such as YAP to be translocated from the nucleus to the cytoplasm where it may reduce cell division, promote apoptosis, or affect the properties of the extra-cellular matrix. Topological defects provide localised points of high compressive stress, and controlling their positions may provide a route to influencing biological functions.

## Electronic supplementary material

Description of Additional Supplementary Files

Supplementary Movie 1
