# Supplementary material for: Active nematics
Source: Nat Commun. 2018 Aug 21;9:3246. doi: 10.1038/s41467-018-05666-8 (PMC6104062; doi:10.1038/s41467-018-05666-8)
Supplement: Supplementary file 1 — Description of Additional Supplementary Files [file 41467_2018_5666_MOESM1_ESM.pdf]

Description of Additional Supplementary Files

File Name: Supplementary Movie 1
Description: Active turbulence and active topological defects in a microtubule/kinesin motor mixture.
